# The Capabilities and Limitations of Clinical Magnetic Resonance Imaging for Detecting Kidney Stones: A Retrospective Study

**DOI:** 10.1155/2016/4935656

**Published:** 2016-11-17

**Authors:** El-Sayed H. Ibrahim, Joseph G. Cernigliaro, Mellena D. Bridges, Robert A. Pooley, William E. Haley

**Affiliations:** ^1^Mayo Clinic, 4500 San Pablo Rd, Jacksonville, FL 32224, USA; ^2^University of Michigan, 1500 E. Medical Center Dr, Ann Arbor, MI 48109, USA

## Abstract

The purpose of this work was to investigate the performance of currently available magnetic resonance imaging (MRI) for detecting kidney stones, compared to computed tomography (CT) results, and to determine the characteristics of successfully detected stones. Patients who had undergone both abdominal/pelvic CT and MRI exams within 30 days were studied. The images were reviewed by two expert radiologists blinded to the patients' respective radiological diagnoses. The study consisted of four steps: (1) reviewing the MRI images and determining whether any kidney stone(s) are identified; (2) reviewing the corresponding CT images and confirming whether kidney stones are identified; (3) reviewing the MRI images a second time, armed with the information from the corresponding CT, noting whether any kidney stones are positively identified that were previously missed; (4) for all stones MRI-confirmed on previous steps, the radiologist experts being asked to answer whether in retrospect, with knowledge of size and location on corresponding CT, these stones would be affirmed as confidently identified on MRI or not. In this best-case scenario involving knowledge of stones and their locations on concurrent CT, radiologist experts detected 19% of kidney stones on MRI, with stone size being a major factor for stone identification.

## 1. Introduction

### 1.1. Nephrolithiasis

Approximately 11% of men and 7% of women are stone formers, and this is an increasingly prevalent problem [1]. The lifetime risk for US adults is estimated to be 1 : 5. Further, recurrent kidney stone formation is very common. Nephrolithiasis is associated with high treatment costs, estimated at over $5 billion per year in the United States [2]. In addition, recent studies showed that nephrolithiasis is associated with a number of diseases, including cardiovascular and cerebrovascular disease, diabetes, obesity, hypertension, and chronic kidney disease [3].

### 1.2. CT Imaging of Kidney Stones

With 90–100% sensitivity and specificity, computed tomography (CT) has been established as the method of choice for imaging kidney stones [4]. Further, the recent introduction of dual-energy CT (DECT) adds the capability of differentiating uric-acid (UA) from non-UA stones, which is important insofar as different treatment strategies may be required for best outcomes [5]. Nevertheless, CT is associated with exposure to ionizing radiation, which is a concern in young patients, pregnant women, and recurrent stone formers. One study found that, on average, kidney stone patients receive about 2.5 CT scans, with 10% of the patients receiving 5 or more scans [6]. Although low-dose CT scanning is gaining traction, it has been recently reported that the median total effective radiation dose per kidney stone patient was 29.7 mSv, while 20% of the patients received total radiation doses greater than 50 mSv [7]. Taken together, we deduce that finding alternative imaging techniques for kidney stones is warranted, especially for the most vulnerable patients.

### 1.3. MR Imaging of the Kidneys

Among various imaging modalities, magnetic resonance imaging (MRI) is characterized by high tissue contrast and spatial resolution, lack of ionizing radiation or radioactive materials, and a large number of imaging parameters that can be adjusted to accentuate the visualization of certain tissue or physiological function. Specifically, for kidney imaging, MRI has the potential of obtaining both functional and anatomical information in the same exam. Currently, noninvasive evaluation of multiple renal function parameters is possible, such as glomerular filtration, tubular concentration, regional perfusion, water movement, and oxygenation [8].

### 1.4. MR Imaging of Kidney Stones

Despite the successes of MRI for anatomical and functional imaging of the kidneys, its role in renal stone imaging has traditionally been limited [9]. Using conventional MR imaging, stones appear as nonspecific signal voids, easily overlooked or confused with other structures or artifacts. As a result of this limitation, guidelines have excluded MRI as a kidney stone imaging modality and radiologists do not attempt to identify stones on MRI images [10].

In this work, we conducted a retrospective study to document the performance of currently available clinical MRI images for detecting kidney stones, compared to gold standard CT, and to determine the characteristics of successfully detected stones.

## 2. Methods

### 2.1. Study Design

In this IRB-approved, retrospective study, patients treated at our institution between 2009 and 2012 who underwent both abdominal/pelvic CT and MRI exams within 30 days were studied. The CT reports of the patients were reviewed to identify those diagnosed with kidney stones. Patients who passed stones or had them extracted during the period between the CT and MR scans were excluded. A total of 160 patients were identified with an interval between the CT and MR examinations of 11 ± 9 days. These cases were reviewed by two radiologists (80 cases each): M. B. (Reviewer 1) and J. C. (Reviewer 2), both highly qualified and experienced fellowship trained experts in abdominal imaging with CT and MRI. The reviewers were blinded to the patients' radiological diagnoses, except that they were aware that all patients had kidney stones noted on CT. The study protocol provided to our radiologist reviewers consisted of four steps: (1) reviewing the MRI images and determining whether any kidney stone(s) are identified, noting size and location; (2) reviewing the corresponding CT images and confirming whether kidney stones are identified, noting size and location; (3) reviewing the MRI images a second time, armed with the information from the corresponding CT, noting whether any kidney stones are positively identified that were previously missed on the first read; (4) for all stones MRI-confirmed on previous steps, on a third review of the MRI images, the radiologist experts being asked to answer yes/no whether in retrospect, with knowledge of size and location on corresponding CT, these stones would be affirmed as confidently identified on MRI or not. At each step, identified kidney stones were characterized by size and location. Interobserver differences were assessed.

### 2.2. Imaging Protocols

The CT images were acquired on Siemens CT scanners (either single-source or dual-source scanners, Siemens Healthcare, Forchheim, Germany) using renal stone imaging protocols. Continuous images were acquired from just above the diaphragm through the pubic symphysis. For patients with a cross-sectional diameter of 35 cm and below, the tube voltages/reference effective tube current-time products were set to 80 kVp/419 mAs and 140 kVp/162 mAs with quality reference CTDIvol = 15.51 mGy. In patients with a cross-sectional diameter greater than 35 cm, the tube voltages/reference effective tube current-time products were set to 100 kVp/210 mAs and 140 kVp/162 mAs with quality reference CTDIvol = 16.61 mGy. For dual-source DECT, other scan parameters were constant regardless of cross-sectional diameter: collimation = 32 × 0.6 mm and pitch = 0.7. Image reconstruction was performed using a mixed (low and high kVp) dataset with 3-mm slice thickness and 2.5-mm slice interval with a standard soft tissue (B30f) convolution kernel. Syngo software (Siemens Healthcare) was utilized to create material-specific chromatic images using 1-mm slice thickness and 0.8-mm slice interval with D30f kernel. In the single-source scans, the tube voltage (kVp) was set to 120 with reference effective tube current-time product of 240 mAs and CTDIvol = 16.18 mGy. Collimation = 24 × 1.2 mm and pitch = 1. Reconstruction was performed using slice thickness and slice interval of 0.5 mm with B30f convolution kernel.

The MRI images were acquired on Siemens MRI scanners (Siemens Healthcare, Erlangen, Germany), with the following imaging parameters: matrix = 177 × 256; resolution = 1.4 × 1.4 mm^2^; slice thickness = 6 mm; flip angle = 150°; echo train length = 256; echo time (TE) = 84 ms; repetition time (TR) = 1200 ms; # of averages = 1; and readout bandwidth = 362 Hz/pixel. All but 8 MRI studies were performed with IV gadolinium, and standard sequences were available for review including HASTE, T1, T2, diffusion, and pre- and postgadolinium fat-saturated T1.

## 3. Results

**Figure 1:**
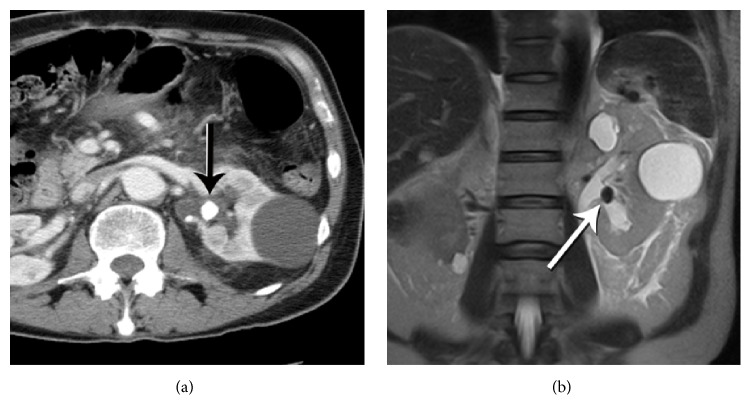
Stone visible on both CT and MR. (a) Enhanced axial CT demonstrates a large stone in the left renal pelvis (arrow). (b) Coronal HASTE MRI image clearly depicts the hypointense stone (arrow) against the backdrop of hyperintense urine.

**Figure 2 fig2:**
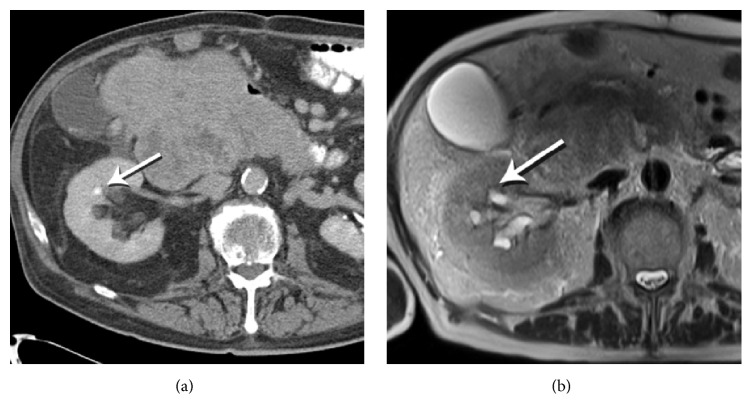
Stone visible on CT, but not on MR. (a) Contrast-enhanced CT performed for tumor staging reveals a stone in a right renal interpolar calyx (arrow). (b) Axial HASTE MRI image fails to detect the stone in the same calyx (arrow).

**Figure 3:**
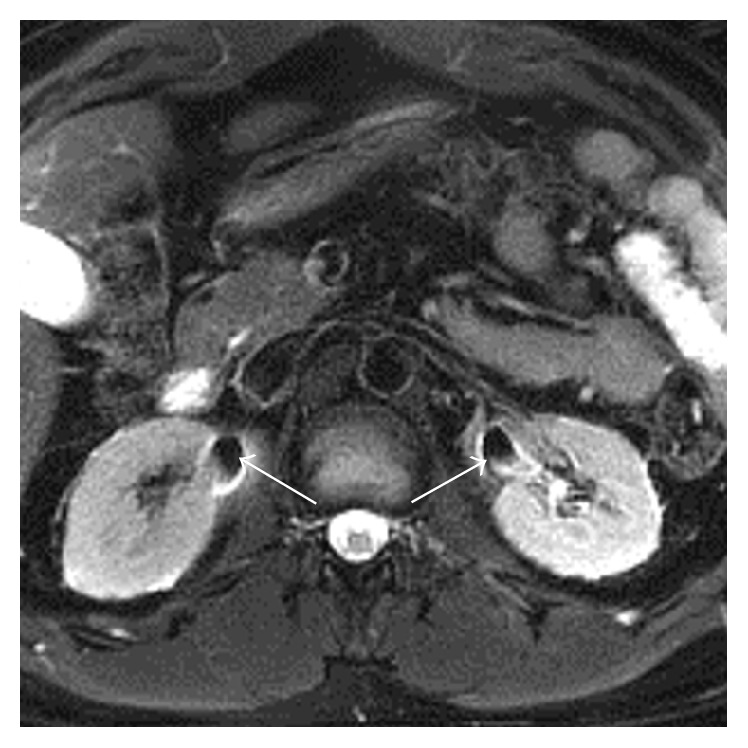
MRI artifacts mimicking kidney stones. An MRI image showing signal void due to T2^*∗*^ artifact from concentrated gadolinium in both collecting systems (arrows). These signal voids could readily be mistaken for stones.

**Figure 4:**
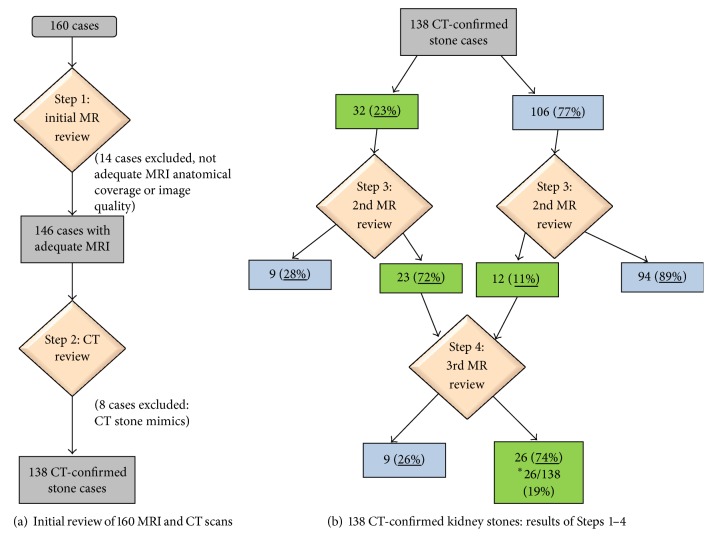
Study design and results. Following Steps  1 and 2, 138 CT-confirmed stones represented the included cases. Green boxes represent number and % detected by MRI at each step; blue boxes represent those not detected by MRI at each step. *∗* = overall final MRI detected rate.

Figures 1 [Fig fig2]–3 show representative CT and MRI images of kidney stones both detected and missed on the MRI images, as well as stone mimics on MRI.  Figure 4 illustrates the organization of the study and provides a flow sheet of the findings. Of the 160 patients (Figure 4(a)), 14 were excluded due to inadequate anatomical coverage or image quality of the MRI images. Eight additional cases were excluded because of the existence of stone mimics in the CT images. The characteristics of the remaining 138 cases were as follows: 84 males and 54 females; age 62 ± 12 years (30–89 years); stone size (based on CT): 6 ± 3 mm (1–19 mm); and stone locations: lower pole (56), mid pole (14), upper pole (33), interpolar (18), renal pelvis (7), bladder (1), and bilateral (9) locations (Table 1, all cases).

Based on the initial review of the MRI images, the stones in 32 cases (23%) were identified (Figure 4(b)), with the following characteristics: stone size = 8 ± 4 mm and locations: lower pole (12), upper pole (8), interpolar (7), renal pelvis (4), and bladder (1) locations (Table 1). The characteristics of the unidentified 106 stones (77%) were as follows: stone size = 5 ± 3 mm and locations: lower pole (44), mid pole (14), upper pole (25), interpolar (11), renal pelvis (3), and bilateral (9) locations. There was a significant (*P* < 0.0001) difference between the sizes of the identified and unidentified stones; larger stones were more likely identified on MRI images. However, for these 32 identified stones, there was no significant (*P* = 0.451) difference between the stone size determined by CT (8 ± 5 mm) and that determined by MRI (8 ± 4 mm).

On the second reading of the MRI images (Step  3, Figure 4(b)), of the 106 stones originally not identified on the first look at the MRI images, 12 stones (11%) were successfully identified with the following characteristics: stone size: 7 ± 2 mm and locations: lower pole (7), upper pole (3), and interpolar (2) locations. The characteristics of the 94 stones (89%) that remained unidentified on the MRI images on this second read were as follows: stone size = 5 ± 2 mm and locations: lower pole (37), mid pole (14), upper pole (22), interpolar (10), renal pelvis (3), and bilateral (8) locations. The difference between the size of the stones that were successfully identified on the second MRI reading and the size of those that remained unidentifiable was significant (*P* = 0.001). Regarding the 32 stones that were originally identified on the first reading of the MRI images, reviewing the CT images along with a second look at the MRI resulted in excluding 9 (28%) (Figure 4(b)) which were determined to be stone mimics (artifacts) with the following characteristics: size: 6 ± 3 mm and locations: lower pole (4), upper pole (3), interpolar (1), and bladder (1) locations. The characteristics of the 23 (72%) stones that were positively identified on MRI at this step were as follows: stone size = 9 ± 4 mm and locations: lower pole (8), upper pole (5), interpolar (5), renal pelvis (4), and bilateral (1) locations. Ultimately, 35 total stones were positively identified on the MRI images, including 12 that were initially not identified as of the first read of the MRI images, plus the 23 that were confirmed on second read (*vide supra* and Table 1).

In Step  4, the reviewers appraised the MRI images a third time and reported that they would, in retrospect, have called 26 (74%) of the 35 MRI-confirmed stones. This yielded a final rate of stone detection by MRI in this study: 26/138 = 19%; those stones had the following characteristics: stone size = 9 ± 4 mm and locations: lower pole (10), upper pole (6), interpolar (6), renal pelvis (3), and bilateral (1) locations. In the cases of the remaining 9 stones (26%), although these had been confirmed on MRI by the reviewers when armed with corresponding CT images at Step  3, the reviewers determined that, in retrospect, these would not be called stones based on this third review of the MRI images. Characteristics of these latter stones were as follows: stone size = 7 ± 3 mm and locations: lower pole (5), upper pole (2), interpolar (1), and renal pelvis (1) locations (Figure 4(b); Table 1). There was no significant difference between stone sizes at Step  4, comparing those called on this final step to those that in retrospect would not be called stones (*P* = 0.1807).

The characteristics of the 138 included cases reviewed by the two reviewers were similar. Reviewer 1 reviewed 64 cases (38 males) with ages of 62 ± 12 years (32–87 years) and Reviewer 2 reviewed 74 cases (46 males) with ages of 62 ± 12 years (30–89 years). The performances of the two reviewers were as follows: Reviewers 1 and 2 identified 27% and 20% of the stones based on the first MRI reading. The sizes of the identified/not identified stones were 8 ± 4/5 ± 3 mm and 8 ± 4/5 ± 2 mm for Reviewers 1 and 2, respectively. The percentages (sizes) of the stones identified by Reviewers 1 and 2 on the second review MRI images were 27% (9 ± 3 mm) and 24% (8 ± 4 mm), respectively. The sizes of the unidentified stones were 5 ± 3 mm and 5 ± 2 mm by the first and second reviewers, respectively. On the final step, third review of MRI images, Reviewers 1 and 2 reported that they would have called 82% and 67% of the 35 total MRI-identified stones, respectively, in retrospect. The sizes of the stones that Reviewers 1 and 2 would have called/not called were 9 ± 3/8 ± 4 and 9 ± 4/6 ± 2 mm, respectively. At all steps, there were no significant differences in performance between the two reviewers in terms of rates or stone size.

## 4. Discussion

The current study reported the capabilities of clinical MR imaging for detecting kidney stones. The results showed that about one-fifth (overall: 19%) of the stones could be confidently detected on clinical MRI images, using modern technology. The stone size and background contrast are known factors for determining kidney stone visibility on MRI [9]. In the present study, the stones detected on MRI were on average 60% larger than those not detected, with significant differences in the stone sizes between the two groups.

Knowing the stone size and location from corresponding CT images at the second MRI review did not result in identification of many of the 106 previously not identified stones; only 12/106 = 11% of the missed stones were subsequently identified (Figure 4(b), Step  3). Important as well, of the 32 identified stones on the initial review of MRI images 9 (9/32 = 28%) were subsequently excluded on the second review as MRI stone artifacts. Such false positive MRI findings may be due to the nonspecific nature of signal void foci on T2-weighted images. After the third review of the MRI images, our expert radiologists' decision to confidently call 26 of the total 35 MRI-identified stones (Figure 4(b), Step  4), even armed with the knowledge of size and location on corresponding CT, reflects the different nature of stone-to-tissue contrast in CT and MRI.

The MRI performance herein reported in identifying large stones, especially in regions where they are surrounded by bright signal (Figure 1(b)), is in agreement with a previous report that noted a size threshold of 1 cm [9]. In the current study, 9 mm was the average size of the detected stones, with the smallest detected size being 4-5 mm. Although just one-fifth of the stones confirmed on gold standard CT were detected confidently from clinical MRI images, recent advances in MRI hardware capabilities and pulse sequence design hold promise for improving MRI detection of kidney stones. Together with the anatomical and functional information about the kidneys that can be obtained with MRI, a complete MRI exam could be available in the future for comprehensive kidney imaging, including scanning for kidney stones. Such exams could be particularly valuable for imaging vulnerable patients, for example, young patients, pregnant women or those with childbearing age, or recurrent stone formers. Further, the capability of imaging kidney stones with MRI could allow for obtaining this information at no extra cost in patients who are undergoing abdominal MRI scans for other diagnoses.

Although the limited capability of MRI for detecting kidney stones has been previously reported, to the best of our knowledge this is the first study that conducted a systematic retrospective analysis of abdominal/pelvis MRI images on a relatively large number of patients with kidney stones against ground truth CT results. The study design along with the interobserver analysis conducted here allowed for quantitatively assessing the capabilities of MRI for detecting kidney stones along with associated characteristics. Although, due to the retrospective nature of the study, we evaluated only standard clinically implemented MRI sequences, future work by our group includes evaluating newly developed sequences, for example, the ultrashort echo time (UTE) technique, which allows for imaging tissues with very short T2 time constants and could be of potential for imaging kidney stones in the future [11]. Although CT is expected to remain the gold standard for kidney stone imaging, the possibility of reliably imaging the stones with MRI would be a valuable alternative for patients with concerns for repeated exposure to ionizing radiation, for example, young patients, pregnant women (or those of childbearing age), and recurrent stone formers.

In conclusion, MRI has the potential for imaging kidney stones, especially medium-to-large stones (mean ± SD = 9 ± 4 mm) with sufficient background contrast. Further study, using newly developed techniques, is under way and promises to improve the ability of MRI to detect kidney stones, which may be useful in vulnerable groups for whom CT scanning with its attendant radiation exposure may not be recommended.

## Figures and Tables

**Table 1:** Stone location by steps.

Location in urinary tract	All CT-confirmed stones	Initial MRI review	2nd MRI review	3rd MRI review
	*N* = 138	*N* = 32	*N* = 35	*N* = 26
Lower pole	56 (5 ± 3 mm)^*∗*^	12 (5 ± 3 mm)	15 (7 ± 3 mm)	10 (8 ± 2 mm)
Mid pole	14 (5 ± 3 mm)	0	0	0
Upper pole	33 (6 ± 4 mm)	8 (10 ± 6 mm)	8 (10 ± 6 mm)	6 (11 ± 6 mm)
Interpolar	18 (6 ± 3 mm)	7 (8 ± 3 mm)	7 (8 ± 3 mm)	6 (8 ± 3 mm)
Renal pelvis	7 (9 ± 3 mm)	4 (9 ± 3 mm)	4 (9 ± 3 mm)	3 (9 ± 4 mm)
Ureter	1 (3 mm)	0	0	0
Bladder	1 (8 mm)	1 (8 mm)	0	0
Bilateral	9 (4 ± 3 mm)	0	1 (10 mm)	1 (10 mm)

^*∗*^Number of stones (size of stones, mean and SD).
